# Gamma Aminobutyric Acid-Enriched Fermented Oyster (*Crassostrea gigas*) Increases the Length of the Growth Plate on the Proximal Tibia Bone in Sprague-Dawley Rats

**DOI:** 10.3390/molecules25194375

**Published:** 2020-09-23

**Authors:** Hyesook Lee, Hyun Hwangbo, Seon Yeong Ji, Min Yeong Kim, So Young Kim, Da Hye Kim, Su Hyun Hong, Su Jeong Lee, Freshet Assefa, Gi-Young Kim, Eui Kyun Park, Joung-Hyun Park, Bae-Jin Lee, You-Jin Jeon, Yung Hyun Choi

**Affiliations:** 1 Anti-Aging Research Center, Dong-eui University, Busan 47340, Korea; 14769@deu.ac.kr (H.L.); hbhyun2003@naver.com (H.H.); 14602@deu.ac.kr (S.Y.J.); ilytoo365@deu.ac.kr (M.Y.K.); 14731@deu.ac.kr (S.Y.K.); believe0402@naver.com (D.H.K.); hongsh@deu.ac.kr (S.H.H.); 2 Department of Biochemistry, Dong-eui University College of Korean Medicine, Busan 47227, Korea; 3 Department of Molecular Biology, Pusan National University, Busan 46241, Korea; 4 Department of Smart Bio-Health, Dong-eui University, Busan 47340, Korea; 5 Department of Pathology and Regenerative Medicine, School of Dentistry, Kyungpook National University, Daegu 41940, Korea; marhaul@naver.com (S.J.L.); fresheta@gmail.com (F.A.); epark@knu.ac.kr (E.K.P.); 6 Department of Marine Life Science, Jeju National University, Jeju 63243, Korea; immunkim@jejunu.ac.kr (G.-Y.K.); youjinj@jejunu.ac.kr (Y.-J.J.); 7 Ocean Fisheries & Biology Center, Marine Bioprocess Co., Ltd., Busan 46048, Korea; pdc327@hanmail.net (J.-H.P.); hansola82@hanmail.net (B.-J.L.)

**Keywords:** fermented oyster, gamma aminobutyric acid, insulin like growth factor-1, recombinant human growth hormone, tibial growth plate

## Abstract

Bone growth during childhood and puberty determines an adult’s final stature. Although several prior studies have reported that fermented oyster (FO) consisting of a high amount of gamma aminobutyric acid can be attributed to bone health, there is no research on the efficacy of FO on growth regulation and the proximal tibial growth plate. Therefore, in this study, we investigated the effect of FO oral administration on hepatic and serum growth regulator levels and the development of the proximal tibial growth plate in young Sprague-Dawley rats. Both oral administration of FO (FO 100, 100 mg/kg FO and FO 200, 200 mg/kg FO) and subcutaneous injection of recombinant human growth hormone (rhGH, 200 μg/kg of rhGH) for two weeks showed no toxicity. Circulating levels of growth hormone (GH) significantly increased in the FO 200 group. The expression and secretion of insulin-like growth factor-1 (IGF-1) and insulin-like growth factor binding protein-3 (IGFBP-3) were enhanced by FO administration. FO administration promoted the expression of bone morphogenic proteins IGF-1 and IGFBP-3 in the proximal tibial growth plate. This positive effect of FO resulted in incremental growth of the entire plate length by expanding the proliferating and hypertrophic zones in the proximal tibial growth plate. Collectively, our results suggested that oral administration of FO is beneficial for bone health, which may ultimately result in increased height.

Academic editors: Alessia Fazio; Pierluigi Plastina

## 1. Introduction

The *Crassostrea gigas* oyster is the most widely cultivated shellfish and industrially important seafood in Asia and Europe because it is easy to grow and spread and is environmentally tolerant [1,2]. In addition, it is well known that oyster meat is a high-quality marine food resource that contains several vitamins and minerals [2]. Numerous studies have reported that oysters and their bioactive peptides have pharmacological benefits including anti-oxidant [3], anti-microbial [4], osteogenic [5,6], anti-inflammatory [7], postprandial blood glucose control [8], and anti-coagulant properties [1].

Fermentation is a natural or controlled food preservation technique that extends shelf-life and improves flavor and nutrition [9]. Several reports have estimated that the biochemical components of oysters changed during fermentation, altering its biological activities [10,11,12]. Je et al. [10] suggested that during oyster sauce fermentation, the major free amino acids increased including taurine, glutamic acid, glycine, leucine, alanine, and lysine. They also reported that peptides isolated from fermented oyster (FO) sauces possessed angiotensin-converting enzyme inhibitory activity, and oral administration of these peptides suppressed blood pressure in spontaneously hypertensive rats [11]. Recently accumulated evidence has established that FO is attributed to bone health [12,13,14,15]. Data published in 2019 demonstrated that FO prevented ovariectomy-induced bone loss and suppressed osteoclastogenesis [13]. A recent study reported that FO promotes bone formation by osteoblast differentiation via activating the Wnt/β-catenin signaling pathway in mouse pre-osteoblast MC3T3-E1 cells, human osteoblast-like MG-63 cells, and zebrafish larvae [14]. Our previous study suggested that FO attenuates osteoclastogenesis via suppression of reactive oxygen species in mouse macrophage cells [15]. We recently demonstrated that fermentation of oyster by *Lactobacillus brevis* BJ20 increased the amount of gamma aminobutyric acid (GABA), which may contribute to increased height in Sprague-Dawley (SD) rats [12]. GABA is associated with bone health and might be a biomarker of osteoporosis diagnosis and therapy [16]. According to a recent report, high levels of serum GABA were observed in young women, which plays a positive role in physical activity, whereas GABA levels were lower in elderly women with osteoporotic fractures [16]. Our previous study suggested that FO has rich GABA content and increases body length and hepatic insulin-like growth factor-1 (IGF-1) synthesis [12]. However, there are limitations that no statistical significance on circulating growth hormone levels and bone morphometric parameters upon oral administration of 100 mg/kg FO. Therefore, the present study assessed the efficacy of high doses of FO in growing rats. We observed the efficacy on growth upon oral administration of 200 mg/kg FO for two weeks in young SD rats. We also investigated the effect of oral FO administration on hepatic and serum growth regulator levels and the development of the proximal tibial growth plate.

## 2. Results

### 2.1. Fermented Oyster (FO) Administration Has No Toxicity

The body weight gains of the FO100 (64.97 ± 5.78 g), FO200 (63.92 ± 1.87 g), and rhGH (70.04 ± 8.70 g) groups were similar to those of the normal group (62.43 ± 8.95 g). Body weight gain was not significantly different between all groups, although body weight gain was slightly increased in the treated groups (data not shown). Meanwhile, the changes in hematological profiles following FO administration were investigated. Red blood cells (RBC), white blood cells (WBC), hematocrit, hemoglobin, mean corpuscular volume (MCV), mean corpuscular hemoglobin (MCH), MCH concentration (MCHC), and platelets showed no differences among all of the groups (Table 1). There was no difference between each group in the values of the biochemical indices including alanine aminotransferase (ALT), aspartate aminotransferase (AST), blood urea nitrogen (BUN), creatinine, and calcium (Table 1).

### 2.2. FO Increased the Expression of Hepatic Insulin-Like Growth Factor-1 (IGF-1) and Insulin-Like Growth Factor Binding Protein-3 (IGFBP-3)

**Figure 1:**
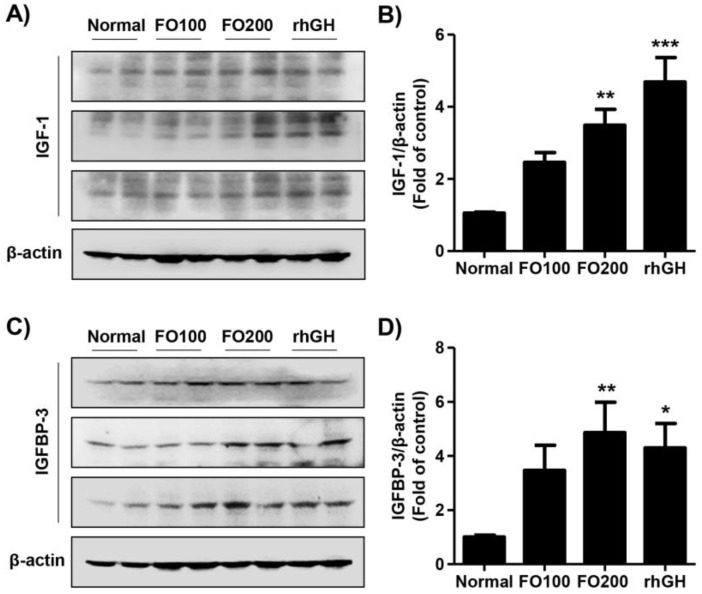
The protein expression of hepatic IGF-1 and IGFBP-3. Liver extract from Sprague-Dawley rats 14 days after treatment were analyzed via western blotting with anti-IGF-1 (**A**) and anti-IGFBP-3 (**C**) antibodies. β-actin served as the loading control. (**B**,**D**) Quantification of IGF-1 and IGFBP-3 levels. Data are expressed as means ± SD (*n* = 6). * *p* < 0.05, ** *p* < 0.01 and, *** *p* < 0.001 vs. normal. IGF-1, insulin-like growth factor-1; IGFBP-3, insulin-like growth factor binding protein-3.

We evaluated the effect of FO administration on the expression of IGF-1 and IGFBP-3 in liver tissue. Analysis of the effects of test substances on hepatic IGF-1 expression showed that the expression significantly increased to 3.86-fold of normal in the FO200 group (Figure 1A,B). The expression of hepatic IGFBP-3 was also markedly increased by FO200 administration (4.49-fold of normal, Figure 1C,D). Administration of FO100 did not affect the expression of IGF-1 and IGFBP-3 in the liver tissue. However, the expression of hepatic IGF-1 and IGFBP-3 were remarkably upregulated by recombinant human growth hormone (rhGH) injection (IGF-1, 4.72-fold of normal; IGFBP-3, 4.15-fold of normal).

### 2.3. FO Induced Circulating Levels of Growth Hormone (GH), IGF-1, and IGFBP-3

**Figure 2:**
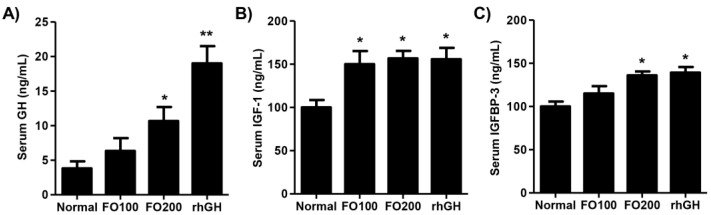
The serum GH (**A**), IGF-1 (**B**), and IGFBP-3 (**C**) levels. Serum from Sprague-Dawley rats 14 days after treatment were analyzed using commercial ELISA kits. Data are expressed as means ± standard deviation (SD, *n* = 6). * *p* < 0.05 and ** *p* < 0.01 vs. normal. GH, growth hormone; IGF-1, insulin-like growth factor-1; IGFBP-3, insulin-like growth factor binding protein-3.

Changes in the serum GH, IGF-1, and IGFBP-3 levels following FO administration were then estimated. As shown in Figure 2A, FO200 administration increased the serum GH levels, which had statistical significance (2.85-fold of normal, *p* < 0.05), and the levels were also elevated by rhGH injection (5.42-fold of normal). The circulating levels of IGF-1 noticeably increased in the FO100 and FO200 administration groups, and the levels were similar to the rhGH group (Figure 2B). The serum IGFBP-3 levels were markedly upregulated by FO200 administration (1.28-fold of normal) and rhGH injection (1.36-fold of normal), but not FO100 (Figure 2C).

### 2.4. FO Increased the Height of the Proximal Tibial Growth Plate

**Figure 3:**
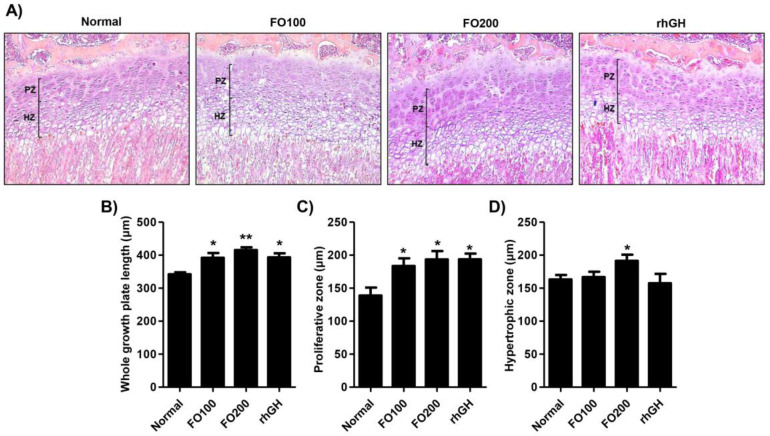
Histology of the proximal tibial growth plate in the Sprague-Dawley rats and height measurements. (**A**) Hematoxylin & eosin (H&E) staining of the proximal tibial growth plate (100× magnification). (**B**) Heights of the entire growth plate were measured on tibial sections stained with H&E. (**C**) Length of the proliferating zone in the growth plates. (**D**) Length of the hypertrophic zone in the growth plates. Heights were measured on tibial sections stained with H&E (100×). Data are expressed as means ± SD (*n* = 6). * *p* < 0.05 and ** *p* < 0.01 vs. normal. PZ, proliferating zone; HZ, hypertrophic zone.

Examination of hematoxylin and eosin (H&E) stained sections of the proximal tibia revealed changes in the growth plate (Figure 3A). Treatment with rhGH (383.79 ± 45.92 μm), FO100 (385.30 ± 40.34 μm), and FO200 (410.41 ± 33.43 μm) significantly increased the growth plate’s total height compared to the vehicle treated normal group (334.69 ± 28.53 μm) (Figure 3B). Detailed measurement of the growth plate demonstrated a significant increase in the height of the proliferation zone in the treated groups compared to the normal group: normal group (138.76 ± 20.72 μm), rhGH (181.19 ± 25.38 μm), FO100 (173.48 ± 17.26 μm), and FO200 (181.54 ± 22.39 μm). As shown in Figure 3C, the height of the hypertrophic zone did not reveal significant differences between the normal (161.12 ± 19.85 μm), rhGH (153.66 ± 30.28 μm), and FO100 (162.58 ± 19.21 μm) treated groups. However, the height of the hypertrophic zone significantly increased in the F0200 (187.52 ± 21.91 μm) treated groups compared to the other groups.

### 2.5. FO Enhanced the Expression of Bone Morphogenetic Proteins in the Proximal Tibial Growth Plate

**Figure 4:**
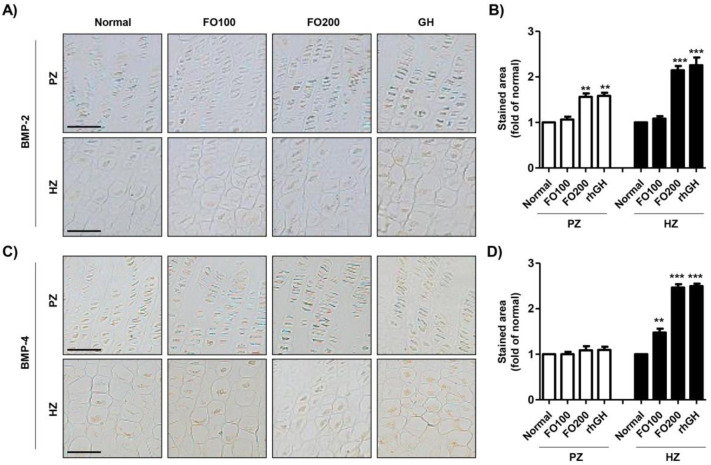
Immunohistochemical localization of bone morphogenic proteins in the proximal tibial growth plate. BMP-2 (**A**) and BMP-4 (**C**) were detected in the proliferating and hypertrophic zone of the proximal tibial growth plate. Scale bar; 50 μm. (**B**,**D**) The stained area of BMP-2 and BMP-4 was analyzed using ImageJ^®^ and calculated in terms of the fold of the control. Data are expressed as means ± SD (*n* = 6). ** *p* < 0.01 and *** *p* < 0.001 vs. normal. BMP-2, bone morphogenetic protein-2; BMP-4, bone morphogenetic protein-4; PZ, proliferating zone; HZ, hypertrophic zone.

We next observed the changes in the expression of bone morphogenic proteins in the tibial growth plate following FO administration. Bone morphogenetic protein (BMP)-2 expression was higher in the hypertrophic zones than in the proliferative zones in the proximal tibial growth plate (Figure 4A,B). The expression of BMP-2 was markedly increased by FO200 administration and rhGH injection. As shown in Figure 4C,D, in the normal group, BMP-4 was more highly expressed in the proliferative zone than in the hypertrophic zone. The expression of BMP-4 in the proliferative zone was not different between the groups. However, the expression of BMP-4 in the hypertrophic zone was markedly enhanced by FO administration in a dose-dependent manner. The rhGH injection induced marked incremental expression of BMP-4 in the hypertrophic zone in the proximal tibial growth plate.

### 2.6. FO Promoted the Expression of IGF-1 and IGFBP in the Proximal Tibial Growth Plate

**Figure 5:**
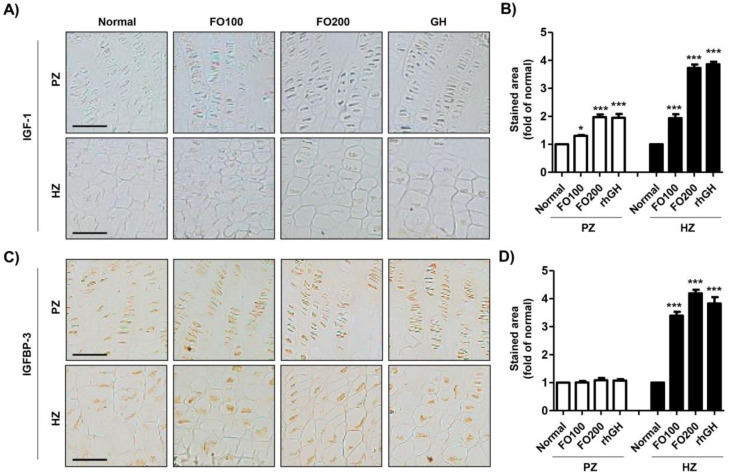
Immunohistochemical localization of IGF-1 and IGFBP-3 in the proximal tibial growth plate. IGF-1 (**A**) and IGFBP-3 (**C**) were detected in the proliferating and hypertrophic zones in the proximal tibial growth plate. Scale bar; 50 μm. (**B**,**D**) The stained area of IGF-1 and IGFBP-3 was analyzed using ImageJ^®^ and calculated in terms of the fold of the control. Data are expressed as means ± SD (*n* = 6). * *p* < 0.05 and *** *p* < 0.001 vs. normal. IGF-1, insulin-like growth factor-1; IGFBP-3, insulin-like growth factor binding protein-3; PZ, proliferating zone; HZ, hypertrophic zone.

To investigate whether FO administration regulated the expression of IGF-1 and IGFBP-3 in the proximal tibial growth plate, we performed additional immunohistochemical analysis. Our results showed that IGF-1 was only slightly expressed in both the proliferative and hypertrophic zones (Figure 5A,B). However, the administration of FO substantially increased IGF-1 expression in both the proliferative and hypertrophic zones, and its increase had a concentration-dependent tendency. In addition, rhGH injection enhanced IGF-1 expression, which was similar to the result in the FO200 group. In the proliferative zone, there was no difference in the expression of IGFBP-3 between the groups (Figure 5B–D). However, the expression of IGFBP-3 in the hypertrophic zone was markedly enhanced by FO administration and rhGH injection.

## 3. Discussion

GABA is a non-protein amino acid that is widely distributed in microorganisms, plants, and animals, and is produced by glutamate decarboxylase that catalyzes the irreversible decarboxylation of L-glutamate to GABA [17,18]. A number of microorganisms of bacteria and fungi have been reported to produce GABA [19]. Among the microorganisms, lactic acid bacteria is the most practical group of bacteria for GABA production, which makes high levels of GABA [19]. Recently, *Lactobacillus brevis* and *Lactococcus lactis* isolated from many fermented foods have been used for the mass production of GABA [20]. A wide range of traditional foods produced by microbial fermentation contain GABA, in which GABA is safe and eco-friendly, and also has the possibility of providing new health-benefited products enriched with GABA [21]. Numerous studies have demonstrated that GABA-enriched functional food have physiological benefits such as suppression of blood pressure [22], hepatoprotection [23], decrease in gut inflammation [24], and regulation of glucose tolerance [25]. Furthermore, several reports have demonstrated that GABA promoted bone health [15,16,26,27,28]. Watanabe et al. [26] reported that GABA directly regulates the muscle tone, and Muhammad et al. [27] suggested GABA promotes osteoblastogenesis by stimulation of bone formation genes. More recently, Jeong et al. [15] found that GABA-enriched seaweed suppressed osteoclastogenesis. Additionally, one study reported that peripheral GABA increases plasma GH concentration in humans [28]. In this regard, our previous finding demonstrated that FO had a high content of GABA by fermentation with *Lactobacillus brevis* BJ20 [12]. Our recent reports suggest that FO prevented osteoclast differentiation, stimulated bone formation, and proposed the possibility of promotion on bone growth [12,13,14]. Based on these previous studies, we considered that the beneficial effect of FO on bone health was associated with a high content of GABA. Therefore, in the present study, we investigated the effect of GABA-enriched FO on hepatic and serum growth regulator levels and the development of the proximal tibial growth plate in SD rats.

The development of the human body is a multifactorial process involving bone tissue accumulation influenced by genetic, nutritional, environmental, and hormonal factors [29]. This process begins during the fetal age and ends in adolescence with the fusion of the epiphyseal growth plate, which determines an individual’s final stature. [30]. Therefore, childhood and pubertal growth has an important impact on height and depends on GH, which has long been established as a longitudinal growth regulator [31,32]. GH is the main hormone involved in growth and has a stimulatory effect on osteoblast precursors and stimulate osteoblast proliferation and activity [32,33]. In the liver, GH accelerates IGF-1 synthesis and subsequently secretion in the blood [33]. GH stimulates the local production of IGF-1 through a direct effect on cartilage cells in the growth plates [34]. IGF-1 is primarily present in a complex form with IGFBP-3, which is synthesized in the liver, and it plays a significant role in growth-plate mediated growth [35,36]. In this respect, numerous reports have established that longitudinal bone growth is stimulated by IGF-1, which has been proposed to play an essential role in bone metabolism [37,38]. Moreover, IGFBP-3 is considered as a biochemically excellent index of GH level due to it is GH dependent and is maintained at a regular daily concentration [39]. Actually, several studies have suggested that the level of IGFBP-3 may be superior to the measurement of IGF-1 in the diagnosis of GH deficiency, and in reflecting actual serum levels of IGF-1 [40,41,42]. In 2016, Lee et al. also demonstrated that oral administration of *Phlomis umbrosa* root increased longitudinal bone growth rate by stimulating proliferation and hypertrophy of chondrocyte with the increment of circulating IGFBP-3 [43]. Furthermore, Kim et al. reported that oral administration of herbal extracts increased the expression of IGF-1 and IGFBP-3 on the growth plate, and most rats had a direct proportional relationship between IGF-1 and IGFBP-3 [44]. Therefore, observations of changes in circulating GH, IGF-1, and IGFBP-3 as well as hepatic IGF-1 and IGFBP-3 expression are important for growth evaluation. In the present study, we evaluated the effect of high doses of FO on growth regulators including GH, IGF, and IGFBP-3 in young SD rats. In accordance with previous studies, our findings showed that the administration of FO 200 mg/kg stimulated the levels of serum GH as well as the expression of IGF-1 in liver and blood (Figure 1 and Figure 2). We found it interesting that in the FO 200 mg/kg group and rhGH group, the IGFBP-3 levels were increased in the liver and blood. In addition, the local expression of IGF-1 and IGFBP-3 was also observed in the hypertrophic zone in the proximal tibial growth plate (Figure 5). These results suggested that oral administration of FO positively regulates circulating and hepatic IGF-1 and IGFBP-3 via incremental GH secretion and has a direct impact on the local proximal tibial growth plate, ultimately contributing to growth.

During childhood, bone mass accretion is a combination of bone remodeling and bone growth [33]. Bone remodeling is the course of new bone formation and resorption by osteoblasts and osteoclasts, respectively [33]. GH regulates both osteoblast proliferation and promotes bone formation, and osteoclast differentiation stimulates bone resorption [45]. Accumulated evidence supports that FO regulates bone remodeling and has a net effect on bone accretion by suppressing osteoclastogenesis and promoting osteogenesis [13,14]. Bone growth primarily occurs at the growth plates and depends on the proliferation and differentiation of chondrocytes [46,47]. The growth plate consists of three principal zones: the resting, proliferative, and hypertrophic zones [48]. The resting zone is adjacent to the epiphyseal bone and maintains the growth plate [49]. The proliferative zone contains replicating chondrocytes arranged in columns parallel to the bone’s long axis [46,47,48]. Terminally differentiated chondrocytes enlarge to become hypertrophic chondrocytes that maintain a columnar alignment in the hypertrophic zone [46,47,48]. In the present study, we investigated the effect of FO on histological changes in the proximal tibial growth plate. Our results showed that FO administration markedly increased the expansion of the proliferative and hypertrophic zones in the growth plate (Figure 3). FO also contributes to increasing the entire growth plate length. Numerous studies suggested that bone growth results in the expansion of the proliferative and hypertrophic zones in growth plates with associated matrix synthesis [48,50]. The oral administration of FO promotes the enlargement of the proximal tibial growth plate, increasing height.

Bone morphogenetic proteins (BMPs) are multifunctional growth factors that play a critical role in bone formation [51]. BMP signal activation is required for undifferentiated mesenchymal cells to become precursors of osteoblasts and chondrocytes [51,52]. Shu et al. reported knockdown of BMP-2 and BMP-4 due to disorganization of chondrocytes, dysregulation of differentiation, and increased apoptosis in the growth plate [53]. Another study showed that BMP-2-deficent mice demonstrated failed fracture healing due to the absence of chondrogenesis at injured lesions [54]. Another study also demonstrated that BMP is required for normal osteogenesis and is a key regulator promoting proper endochondral bone formation [55]. In line with these findings, our results showed that FO promoted the expression of BMP-2 and BMP-4 in the hypertrophic zone in the proximal tibial growth plate. Based on this result, oral administration of FO upregulates BMP expression in the growth plate and may contribute to bone health via bone formation.

**Figure 6:**
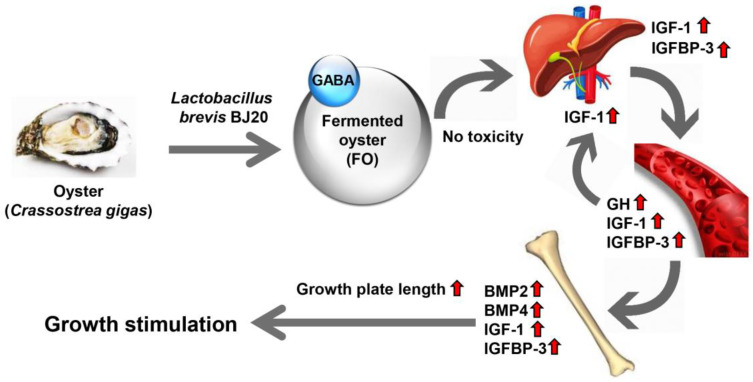
GABA-enriched FO promoted the length of the growth plate on the longitudinal bone in the Sprague-Dawley rats.

In summary, administration of GABA-enriched FO has no toxicity and markedly increased the hepatic expression and circulating levels of GH, IGF-1, and IGFBP-3. Administration of FO substantially upregulated the expression of BMPs, IGF-1, and IGFBP-3 in the hypertrophic zone of the proximal tibial growth plate. These effects of FO contribute to increasing the length of the entire growth plate in the proximal tibia, which may ultimately result in increased height (Figure 6).

## 4. Materials and Methods

### 4.1. Preparation of Fermented Oyster

FO extract was obtained from Marine Bioprocess Co. Ltd. (Busan, Korea). In brief, the FO used in this study was oyster fermented with *L. brevis* BJ20, and glutamic acid and dextrin were used on behalf of glutamate and an excipient, respectively. Glutamate was used as a precursor to produce GABA through a decarboxylation reaction during fermentation with *L. brevis* BJ20 [56]. In the present study, used FO was the same batch as used that in a previous report [12], which was comprised of 46 g/100 g carbohydrate, 36 g/100 g crude protein, 6.3 g/100 g sugars, and 114 mg/g GABA. FO was diluted with distilled water immediately before use.

### 4.2. Animal Study

Female Sprague-Dawley rats (three weeks old) were purchased from Koatech Laboratory Animals, Inc. (Pyeongtaek, Gyeonggi, Korea) and adapted for one week. All of the procedures were followed in accordance with the Guide for the Care and Use of Laboratory Animals and approved by the Institutional Animal Care and Use Committee of Dong-eui University (No. R2019–002). Thirty-two rats were randomly assigned to four groups of eight rats: group 1 (normal, 100 μL of distilled water), group 2 (100 μL of 100 mg/kg FO), group 3 (100 μL of 200 mg/kg FO), and group 4 (subcutaneous injection of 200 μg/kg rhGH; Growtropin-II Dong-A ST Co. Ltd., Seoul, Korea). Groups 1, 2 and 3 were orally administrated directly into the stomach of rats via metal gavage needles (Braintree Scientific, Braintree, IL, MA), while Group 4 was subcutaneously injected. All of the treatments were administered once a day in the morning for two weeks. The body wight was measured weekly. All of the rats were given ad libitum access to standard chow and water and sacrificed on day 14 of the study.

### 4.3. Collection of Blood and Tissue Samples

Blood samples were collected from the heart using BD Vacutainer ethylenediaminetetraacetic acid (EDTA)-containing tubes (Becton Dickinson, Franklin Lakes, NJ, USA). Whole blood samples were used for hematological analysis. Serum samples that were obtained by centrifugation at 3000 g for 10 min were stored at −80 °C for later biochemical and ELISA analyses. After perfusion, liver samples were immediately surgically excised and stored at −80 °C for western blotting. Longitudinal bone was dissected, fixed 10% formalin overnight, and decalcified for one month in 0.5 M EDTA (pH 7.4) at 4 °C with constant shaking. Tissues were dehydrated by passage through an ethanol series, cleared three times in xylene, embedded in paraffin, and sectioned to 5 μm with a microtome (Leica Biosystems, Nussloch, Germany). Paraffin sections of the proximal tibia then underwent histochemical and immunohistochemical analysis.

### 4.4. Hematological and Biochemical Analysis

The hematological analyses were conducted using a Sysmex XN-9000 analyzer (Sysmex Corporation, Kobe, Japan) to assess the complete blood count: RBC, WBC, hematocrit, hemoglobin, MCV, MCH, MCHC, and platelet counts. The biochemical analysis was validated using a Cobas 8000 C702 chemistry analyzer (Roche, Mannheim, Germany). Components including ALT, AST, BUN, creatinine, and calcium were detected as previously described [57].

### 4.5. Western Blotting Analysis

Western blotting analysis was conducted as previously described [12]. In brief, the liver tissues were lysed in radioimmunoprecipitation assay lysis buffer, and the total protein concentration was determined using Bradford Protein Assay Kits (Bio-Rad Laboratories, Hercules, CA, USA). A total of 50 μg of protein was separated on 15% sodium dodecyl sulfate-polyacrylamide gel electrophoresis gels and transferred to polyvinylidene fluoride (PVDF) membranes after electrophoresis. The membranes were then blocked with 5% non-fat dry milk in Tris-buffered saline containing 0.1% Triton X-100 (TBST) for 1 h and probed for overnight at 4 °C using primary antibodies: IGF-1 (1:500, sc-74116, Santa Cruz Biotechnology, Santa Cruz, CA, USA), IGFBP-3 (1:500, sc-374365, Santa Cruz Biotechnology), and β-actin (1:2000, sc-47778, Santa Cruz Biotechnology). The PVDF membrane was washed with Tris-buffered saline (TBS) and incubated with appropriate secondary antibody (sc-2005, Santa Cruz Biotechnology) for 1 h at room temperature, then exposed with an enhanced chemiluminescence kit (GE Healthcare Life Sciences Ltd., Amersham Place, Little Chalfont, UK) at the Core-Facility Center for Tissue Regeneration, Dong-eui University (Busan, Korea). Immunoreactive proteins were scanned with a Fusion FX Image system (Vilber Lourmat, Torcy, France). The band density was normalized according to the expression of β-actin as a loading control.

### 4.6. Enzyme-Linked Immunosorbent Assay

Serum GH, IGF-1, and IGFBP-3 levels were measured using an enzyme-linked immunosorbent assay (ELISA), in accordance with the manufacturer’s instructions. A rat GH solid-phase sandwich ELISA kit (KRC5311) was purchased from Thermo Fisher Scientific (Waltham, MA, USA). Quantitative sandwich ELISA kits for mouse IGF (OKBB00165) and mouse IGFBP-3 (OKBB00172) were obtained from AVIVA Systems Biology (San Diego, CA, USA).

### 4.7. Histochemical Analysis

Histochemical analysis of the longitudinal bone was performed using a previously described protocol with slight modifications [15,58]. Paraffin sections of the proximal tibia were dewaxed, rehydrated, and stained with hematoxylin and eosin (Sigma-Aldrich Chemical Co., St. Louis, MO, USA) and representative images were captured using a Leica DM 2500 (Leica Biosystems, Nussloch, Germany). Regions along the center of the growth plates and proliferative and hypertrophic zones were selected for measurements. The height measurements were obtained by using iSolution software (Daejeon, Korea) at 100× magnification. At least 10 measurements were obtained for each section.

### 4.8. Immunohistochemistry

Immunohistochemistry of the longitudinal bone was performed as previously described [59]. Paraffin sections of the proximal tibia were dewaxed, rehydrated, cooked in antigen retrieval buffer (Abcam Inc., Cambridge, UK) for 10 min, and then incubated using a Vectastain ABC kit (Vector Laboratories, Burlingame, CA, USA) for 30 min. The tissue sections were blocked in 5% bovine serum albumin for 1 h and then probed overnight at 4 °C with primary antibodies: BMP-2 (1:200, ab14933, Abcam), BMP-4 (1:200, ab39973, Abcam), IGF-1 (1:200, sc-74116, Santa Cruz Biotechnology), and IGFBP-3 (1:200, sc-374365, Santa Cruz Biotechnology). The sections were then applied with appropriate secondary antibodies for 1 h at room temperature and incubated in ABC reagent for 1 h. Immunoreactions were visualized with 3,3′-diaminobenzidine (DAB) peroxidase substrate and counterstained with Mayer’s hematoxylin (Sigma-Aldrich Chemical Co.). Images of the sections were photographed with a Leica DM 2500. The quantitative analysis of histological staining for BMP-2, BMP-4, IGF-1, and IGFBP-3 was performed using the “threshold tool” of ImageJ^®^ [60].

### 4.9. Statistical Analysis

The results were presented as means ± standard deviation (SD). All of the data were analyzed via one-way ANOVA with Tukey’s post-hoc test (GraphPad Prism 5.03, GraphPad Software, Inc., La Jolla, CA, USA), and *p* < 0.05 was considered statistically significant.

## 5. Conclusions

Collectively, our results suggest that oral administration of FO can lengthen the proximal tibial growth plate and increase growth hormone and growth regulator in the liver and blood. FO’s effect on proximal tibial growth may be due to GABA. Although our findings demonstrated the enhancing effect of FO on the development of young rats, further studies are necessary to confirm the effect of FO on bone micro-architecture and identify the mechanism of action. More evidence is needed to demonstrate the potential benefits on childhood bone growth using clinical trials.

## Figures and Tables

**Table 1:** Hematological and biochemical analysis of the Sprague-Dawley rats 14 days after treatment.

Parameter (Units) ^2^	Group ^1^			
	Normal	FO100	FO200	rhGH
RBC (106/μL)	6.83 ± 0.42	6.77 ± 0.40	6.81 ± 0.37	6.84 ± 0.33
WBC (103/μL)	3.44 ± 0.38	3.52 ± 0.59	3.68 ± 0.64	3.15 ± 0.76
Hematocrit (%)	50.11 ± 2.32	49.74 ± 2.08	51.00 ± 1.93	49.93 ± 2.15
Hemoglobin (g/dL)	14.55 ± 0.74	14.36 ± 0.49	14.64 ± 0.63	14.37 ± 0.58
MCV (fL)	72.68 ± 1.83	72.33 ± 1.54	73.41 ± 1.92	73.51 ± 1.75
MCH (pg)	21.42 ± 0.53	21.04 ± 0.44	20.79 ± 0.67	21.28 ± 0.53
MCHC (g/dL)	28.97 ± 0.36	28.35 ± 0.42	28.75 ± 0.39	28.72 ± 0.35
Platelet (103/μL)	1217.49 ± 236.81	1334.52 ± 201.57	1294.63 ± 194.68	1315.44 ± 186.37
ALT (U/L)	13.72 ± 1.02	13.44 ± 0.85	12.96 ± 0.78	13.35 ± 0.89
AST (U/L)	24.38 ± 2.72	21.06 ± 2.47	22.41 ± 3.11	20.85 ± 3.26
BUN (mg/dL)	19.15 ± 2.34	20.27 ± 2.75	18.89 ± 3.06	18.12 ± 2.51
Creatinine (mg/dL)	0.29 ± 0.04	0.29 ± 0.04	0.29 ± 0.02	0.28 ± 0.04
Calcium (mg/dL)	12.84 ± 0.65	12.37 ± 0.39	12.27 ± 0.41	12.33 ± 0.43

Results are expressed as means ± SD of eight rats in each group. ^1^ Normal distilled water was orally administered; FO100, 100 mg/kg/day of fermented oyster (FO) extract was orally administered; FO200, 200 mg/kg/day of FO was orally administered; rhGH, 200 μg/kg/day of recombinant human growth hormone (rhGH) was subcutaneously injected. ^2^ RBC, red blood cells; WBC, white blood cells; MCV, mean corpuscular volume; MCH, mean corpuscular hemoglobin; MCHC, MCH concentration; ALT, alanine aminotransferase; AST, aspartate aminotransferase; BUN, blood urea nitrogen.
